# Stealth lipid polymer hybrid nanoparticles loaded with rutin for effective brain delivery – comparative study with the gold standard (Tween 80): optimization, characterization and biodistribution

**DOI:** 10.1080/10717544.2017.1410263

**Published:** 2017-12-01

**Authors:** Rania A. H. Ishak, Nada M. Mostafa, Amany O. Kamel

**Affiliations:** a Department of Pharmaceutics and Industrial Pharmacy, Faculty of Pharmacy, Ain Shams University, Cairo, Egypt;; b Department of Pharmacognosy, Faculty of Pharmacy, Ain Shams University, Cairo, Egypt

**Keywords:** Brain delivery, Tween 80, TPGS, Solutol HS 15, lipid polymer hybrid nanoparticles

## Abstract

The blood–brain barrier is considered the leading physiological obstacle hindering the transport of neurotherapeutics to brain cells. The application of nanotechnology coupled with surfactant coating is one of the efficacious tactics overcoming this barrier. The aim of this study was to develop lipid polymer hybrid nanoparticles (LPHNPs), composed of a polymeric core and a phospholipid shell entangled, for the first time, with PEG-based surfactants (SAA) viz. TPGS or Solutol HS 15 in comparison with the gold standard Tween 80, aiming to enhance brain delivery and escape opsonization. LPHNPs were successfully prepared using modified single-step nanoprecipitation technique, loaded with the flavonoid rutin (RU), extracted from the flowers of *Calendula officinalis* L., and recently proved as a promising anti-Alzheimer. The effect of the critical process parameters (CPP) *viz.* PLGA amount, *W*_lecithin_/*W*_PLGA_ ratio, and Tween 80 concentration on critical quality attributes (CQA); entrapment, size and size distribution, was statistically analyzed *via* design of experiments, and optimized using the desirability function. The optimized CPP were maintained while substituting Tween 80 with other PEG-SAA. All hybrid particles exhibited spherical shape with perceptible lipid shells. The biocompatibility of the prepared NPs was confirmed by hemolysis test. The pharmacokinetic assessments, post-intravenous administration to rats, revealed a significant higher RU bioavailability for NPs relative to drug solution. Biodistribution studies proved non-significant differences in RU accumulation within brain, but altered phagocytic uptake among various LPHNPs. The present study endorses the successful development of LPHNPs using PEG-SAA, and confirms the prospective applicability of TPGS and Solutol in enhancing brain delivery.

## 1. Introduction

Neurotherapeutics are classes of drugs used in the treatment of brain or central nervous system disorders. The effectiveness of these actives is generally compromised due to failure reaching the site of action sufficiently. The blood–brain barrier (BBB) is considered the most dominant physiological barrier impeding the passage of neuropharmaceuticals to the brain cells. It is a highly fortified membrane system, composed of specialized capillary endothelial cells, that protects the brain from extraneous organisms and harmful chemicals, and supplies the brain with the nutrients required (Salunkhe et al., 2015). Overcoming the difficulty in crossing BBB is the key strategy for efficient delivery of therapeutic molecules to the brain.

Several delivery approaches were implemented aiming to target drugs to the brain *via* enhancing their transport across BBB. The application of nanotechnology coupled with surfactant coating is one of these propitious tactics.

For a long time, nanoparticle (NP)-mediated drug transport to the brain has been governed by particle coating with surfactants. The surfactant Tween^®^ 80 (polyethylene glycol sorbitan monooleate) is considered the gold standard effectively crossing BBB. The fact is due to the preferential adsorption of apolipoprotein E (Apo E), present in blood, on NP surfaces coated with Tween 80, rendering particles resembling the low density lipoproteins (LDL), hence interacting with LDL receptors on BBB and enhancing their cellular uptake *via* receptor-mediated transcytosis mechanism (Gessner et al., 2000; Göppert & Müller, 2003). Other surfactants were investigated for their capabilities to transport drugs across BBB, from which d-α-Tocopherol polyethylene glycol 1000 succinate (TPGS) and Solutol^®^ HS 15 (polyethylene glycol-15-hydroxy stearate) (Lamprecht & Benoit, 2006; Wa Kasongo, 2010; Agrawa et al., 2017; Meng et al., 2017). Both surfactants count on the inhibition of P-glycoprotein (P-gp) efflux pump, a membrane transporter of the ATP-binding cassette (ABC) superfamily located within BBB, which plays a substantial role in limiting the permeability of many therapeutic agents to the brain. This inhibition relies on an intermingling of membrane fluidization, ATP depletion, and hindrance of substrate binding (Hoosain et al., 2015). A recent publication demonstrated an additional brain targeted mechanism for Solutol HS, analogous to Tween 80, *via* a preferential adsorption of Apo E onto the surface of nanocarriers (Kasongo et al., 2011).

A variety of nanocarriers has been studied for delivering therapeutics to the brain, for instance, polymer-based, dendrimers, micelles and lipid-based carriers (Gabal et al., 2014). The biocompatibility and biodegradability of these carriers have to be guaranteed. Liposomes, phospholipid-based vesicles, are one of the lipid nanocarriers extensively studied for delivering drugs to the brain (Salama et al., 2012a,b), thanks to their lipophilic nature mimicking biological membranes, tending to cross BBB naturally. Although the benefits of these carriers, e.g. the low toxicity and the ability to encapsulate drugs with different properties, they suffer from some limitations; among these are the instability during storage and the loss of their content due to poor structural integrity (Maurer et al., 2001). Polymeric NPs are also considered attractive therapeutic delivery systems widely investigated for brain targeting. They are fabricated from either natural or synthetic polymer, for instance, chitosan and poly-lactide-*co*-glycolide (PLGA), respectively. They exhibit a number of advantages compared with liposomes; the extended stability upon storage, the robust structural integrity and the controlled release pattern for encapsulated drugs (Peer et al., 2007). Based on their hydrophobic characters and/or highly charged surfaces, both nanosystems, i.e. liposomes and polymeric NPs, lack the long circulation property in blood owing to the fast recognition and clearance by the rediculoendothelial system (RES). Hence, PEGylation of these carriers is crucial as it would enhance their bioavailability by prolonging the *in vivo* circulation period (Torchilin, 2005; Sheng et al., 2009).

Aiming to process the limitations of both liposomes and polymeric NPs, a new generation of drug delivery system, so-called lipid polymer hybrid nanoparticles (LPHNPs), was developed combining the characteristics of both systems. These platforms are composed of a polymeric core surrounded by a self-assembly phospholipid shell intertwined with a PEG-containing substrate, typically a PEG-lipid warranting the protracted circulation time (Hadinoto et al., 2013). Even though the benefits of these nanostructures, to our best knowledge, little research has been addressed their application in brain targeting (Mohamed et al., 2014; Agrawal et al., 2015; Shi et al., 2015).

Amongst brain maladies, Alzheimer’s disease (AD) is the most common neurodegenerative disorder causing dementia among the elderly population in the world. It is usually manifested by a bundle of disruptions in memory, thinking, understanding, learning abilities, linguistics, calculation and coordination (Salomone et al., 2012). The impairment in mitochondrial functions in brain cells, expressed by severe reduction in mitochondrial enzyme activities and defects in electron transport, is considered the underlying etiology of AD (Moreira et al., 2007; Young et al., 2010). The oxidative stress allied with an increase in reactive oxygen and nitrogen species (RONS) production and the potentiation in anti-amyloid (Aβ) deposition are additional factors (Omar et al., 2017). Currently, cholinesterase inhibitors and N-methyl-D-aspartate receptor antagonists are the only FDA-approved drug classes for AD treatment. These medications are effective in relieving symptoms for short-duration (one to three years), just at the early stages of the disease and for a restricted number of patients (Salomone et al., 2012).

Nowadays, there is an increased attentiveness coupled with experimental proofs for the potential utility of natural extracts in AD treatment. Polyphenols, alkaloids, terpenoids are among the most promising phyto-constituents interrelated to improve AD symptoms. Amongst the polyphenol-based active structures is the flavonoid rutin (RU), a novel promising candidate reported to exert a boosting anti-AD effect. RU (quercetin-3-*O*-rutinoside), the glycoside of the flavonol quercetin, is found in many plants, for instance, citrus fruits (lemon, orange, and grapefruit), apples, berries, peaches and green tea (Manach et al., 2004). It was reported that RU would exert an anti-oxidant effect via a free radical scavenging property owing to the great number of hydroxyl groups present in its structure. It was also testified its capability to reduce lipid peroxidation and RONS generation in brain tissue homogenates (Gao et al., 2002). Moreover, it was proven to prevent the formation of Aβ25–35 fibrils, reduce Aβ42-induced cytotoxicity in neuroblastoma cells (Wang et al., 2012), and ameliorate spatial memory and cognitive impairments by attenuating oxidative stress and neuro-inflammation in rat models (Pu et al., 2007; Javed et al., 2012).

In the present work, a novel attempt was studied targeting the anti-AD polyphenol drug (RU) to the brain via the innovative nanoconstructs ‘LPHNPs’ coupled with surfactant coating. As a new approach, the classical PEG-lipid component in LPHNPs was substituted, for the first time, with PEG-based surface active agents (SAA) aiming to afford equivalent stealth character to the hybrid particles. Among these SAA, Tween 80, TPGS and Solutol HS 15 are prototypes. These SAA also combined the reported persuasive roles in enhanced brain delivery. In this context, RU was first extracted from the flowers of *Calendula officinalis* L., and then loaded into LPHNPs using design of experiments (DoE). The results were statistically analyzed and then optimized through the desirability function parameter. A comparative study was then developed to compare different coating materials in terms of characterization, biocompatibility, pharmacokinetic and biodistribution studies.

## 2. Materials and methods

### 2.1. Materials

#### 2.1.1. Plant material for RU extraction

The edible flowers of *C. officinalis* L., family Asteraceae were collected from the Botanical Garden of El-Orman, Giza, Egypt. The plant was authenticated by Mrs. Trease Labib, Plant Taxonomy Consultant at the Ministry of Agriculture. A voucher specimen has been deposited at Pharmacognosy Department, Faculty of Pharmacy, Ain Shams University, Abbassiah, Cairo, Egypt (PHG-P-CO-1). All used solvents used for extraction were of high analytical grade.

#### 2.1.2. Formulation materials

Poly-dl-lactide-*co*-glycolide (PLGA) (Purasorb^®^ PDLG 7507; with a molar ratio of 75/25 and an inherent viscosity midpoint of 0.7 dl/g) was gently provided from Corbion Purac Biomaterials (Amsterdam, The Netherlands). The lecithin, Soybean phosphatidylcholine (SPC, Lipoid^®^ S100) was kindly supplied by Lipoid GmbH (Ludwigshafen am Rhein, Germany). TPGS was generously delivered by Isochem (Vert-le-Petit, France). Tween^®^ 80 and Solutol^®^ HS 15 were obtained from Sigma-Aldrich, Chemical Co. (Steinheim, Germany). Methanol (HPLC grade) was purchased from Fluka (Buchs, Switzerland). Potassium dihydrogen phosphate, disodium hydrogen phosphate, sodium chloride, potassium chloride (KCl), acetone, dimethyl sulfoxide (DMSO) and ortho-phosphoric acid were obtained from Adwia, El-Nasr Pharmaceutical Co. (Egypt). Spectra/Por^®^ dialysis membrane, 12,000–14,000 MWCO was purchased from Spectrum Medical Industries (Houston, TX, USA). Nanosep^®^ centrifuge tubes, fitted with an ultra-filter of 100 kDa MWCO, were provided from Pall Life Sciences (East Hills, NY, USA). Deionized water provided from Milli-Q Gradient A10 System was employed all over the research study. All other chemicals and reagents were of analytical grade.

### 2.2. Methods

#### 2.2.1. Extraction, isolation and identification

The marigold (*C. officinalis* L.) flowers (2 kg) were dried in shade, and extracted with neat methanol several times till complete exhaustion. The methanol in the combined extracts was distilled off at 50 °C in a rotary evaporator. The remaining concentrated extract was then subjected to preparative TLC using the BAW solvent system butanol: acetic acid: water (4:1:5, upper layer). A major band showing a dark purple color upon using UV lamp at 365 nm and quenched the UV light at 254 nm, was scratched, dissolved in methanol and the solvent was evaporated *in vacuo* at 50 °C, to obtain a yellow powder that was subjected to UV and NMR spectroscopic analysis. ^1^H and ^13^C-NMR spectroscopic data were measured at 400 and 100 MHz, respectively, in DMSO on a Bruker Ascend-400 spectrometer (Avance BioSpin Inc., Rheinstetten, Germany). The chemical shifts (recorded in ppm) were measured using tetramethyl silane (TMS) as internal standard. The UV spectroscopic measurement was performed on UV-Spectrophotometer (Jasco V630, Tokyo, Japan).

#### 2.2.2. LPHNPs preparation

Lipid-polymer hybrid NPs were prepared using a modified single-step nanoprecipitation technique. Briefly, PLGA, SPC, and drug were all dissolved in 5 ml acetone. RU/PLGA weight ratio was maintained at 1/20 in all prepared formulations. The surfactant (Tween 80) was dispersed in 10 ml deionized water heated to 65 °C. The resulting PLGA/SPC/RU organic solution was then added into the preheated SAA solution dropwise under gentle stirring. The mixed solution was then agitated vigorously for 5 min. followed by gentle stirring for 2 h at room temperature to ensure complete evaporation of the organic solvent. The drug-loaded PLGA NPs were also prepared, for comparison purpose, using the same method described above except lecithin was excluded from the preparation.

#### 2.2.3. Design of the experiments

A response surface methodology (RSM) using two-level full factorial design was implemented aiming to optimize the critical process parameters (CPP) of LPHNPs. The experiment studied the effect of three CPP, namely PLGA amount (*X*_1_), *W*_lecithin_/*W*_PLGA_ ratio (*X*_2_) and Tween 80 concentration (*X*_3_), each at two levels; 50 and 100 mg, 1/1 and 3/1, 0.5 and 1%, respectively. The critical quality attributes (CQA) considered were *Y*_1_; the percent of drug entrapment efficiency (EE), *Y*_2_; the particle size (PS) and *Y*_3_; the polydispersity index (PDI) of the formed NPs. The typical statistical design of the experiment is displayed in Table 1.

**Table 1.** The measured responses of RU-loaded LPHNPs formulations prepared based on two-level full factorial design.

	Factors			Measured responses^a^		
Runs	*X*_1_	*X*_2_	*X*_3_	*Y*_1_: EE (%) ± SD	*Y*_2_: PS (nm) ± SD	*Y*_3_: PDI ± SD
1	50 (−1)	1:1 (−1)	0.5 (−1)	35.45 ± 0.85	288.80 ± 4.66	0.190 ± 0.017
2	100 (+1)	1:1 (−1)	0.5 (−1)	46.90 ± 4.17	256.00 ± 2.97	0.354 ± 0.016
3	50 (−1)	3:1 (+1)	0.5 (−1)	50.55 ± 2.02	183.90 ± 10.46	0.158 ± 0.061
4	100 (+1)	3:1 (+1)	0.5 (−1)	83.15 ± 3.68	333.85 ± 16.62	0.424 ± 0.006
5	50 (−1)	1:1 (−1)	1 (+1)	31.35 ± 1.12	209.50 ± 3.66	0.171 ± 0.024
6	100 (+1)	1:1 (−1)	1 (+1)	35.85 ± 2.32	288.30 ± 2.91	0.343 ± 0.034
7	50 (−1)	3:1 (+1)	1 (+1)	42.59 ± 2.67	147.60 ± 2.32	0.153 ± 0.020
8	100 (+1)	3:1 (+1)	1 (+1)	77.65 ± 2.57	330.80 ± 12.52	0.366 ± 0.008
–	50	0:1	1	17.83 ± 1.05	220.10 ± 2.80	0.069 ± 0.015

*X*_1_: PLGA amount (mg); *X*_2_: *W*_lecithin_/*W*_PLGA_ ratio; *X*_3_: Tween 80 concentration (%w/v); SD: standard deviation.

^a^ Average of three determinations.

N.B.: The last non-numbered experiment represents PLGA NPs (without lecithin) prepared by 50 mg polymer and stabilized with 1% Tween 80, for comparison purpose only.

#### 2.2.4. Recovery and purification of RU-loaded LPHNPs

The prepared hybrid NPs were recovered and purified *via* the dialysis technique using Spectra/Por^®^ dialysis membrane. Different dialysis times; 0.5, 1, 2 and 3 h, were tried and then the purified particles were evaluated in terms of drug EE%, PS, PDI, and ZP.

#### 2.2.5. Characterization of the prepared RU-loaded LPHNPs

##### 2.2.5.1. Determination of RU EE

To determine EE of RU, LPHNPs loaded with RU were separated from the aqueous suspension medium using a cooling microcentrifuge (Hermle Labortechnik GmbH; Model Z216 MK, Germany). A sample of 50 µl of NP dispersion was diluted with 450 µl deionized water, placed in the upper part of a Nanosep^®^ and then subjected to centrifugation at 7000 rpm, 4 °C for 1 h. The filtrate was then collected to determine the amount of un-entrapped RU using HPLC at *λ*_max_ 360 nm. The EE of RU in LPHNPs was calculated according to the following equation:

$$\text{EE}\;\text{(\%)}=\frac{W_{\text{t}}-W_{\text{f}}}{W_{\text{t}}}\times 100$$

where *W*_t_ is the total amount of drug used in the preparation and *W*_f_ is the amount of free drug in the filtrate.

##### 2.2.5.2. PS and PDI analysis

Particle size and polydispersity index measurements were accomplished using dynamic light scattering (DLS) technique via Zetasizer Nano ZS instrument (Malvern Instruments Ltd., Malvern, UK). The PS and PDI of three independent samples were measured in disposable cuvettes at a temperature of 25 ± 0.5 °C after 50-fold dilution with deionized water.

##### 2.2.5.3. Surface charge measurement

Laser Doppler anemometry (LDA) technique was applied for zeta potential (ZP) measurements using Zetasizer Nano ZS instrument (Malvern Instruments Ltd., Malvern, UK). Three independent samples were suitably diluted with 10 mM KCl and then placed in zeta cells for measurement at a temperature of 25 ± 0.5 °C.

##### 2.2.5.4. Particle morphology using high resolution-transmission electron microscope (HR-TEM)

The imaging of the selected RU-loaded LPHNPs, as well as PLGA NPs, was performed *via* HR-TEM (JEOL JEM-2100, Japan) with an acceleration voltage at 200 kV. All samples for TEM imaging were initially prepared by allowing a single drop of NP suspension to dry at room temperature on a carbon-coated copper meshwork after being stained with 1% phosphotungstic acid.

##### 2.2.5.5. Differential scanning calorimeter (DSC) examination

The thermal properties of different samples of RU, PLGA, SPC and selected loaded NP formulations were studied using a differential scanning calorimeter (DC-60 plus; Shimadzu, Japan). The NP formulations were left to dry overnight in a desiccator. Powdered samples were sealed in aluminum pans with lids and heated from 25 to 300 °C at a rate of 10°C/min. under nitrogen flow at a rate of 25 ml/min.

#### *2.2.6. In vitro* drug release study

*In vitro* release experiments of RU from loaded LPHNPs were performed in PBS (pH 7.4) for 12 h. An aliquot of NPs, equivalent to 1 mg RU, was introduced in a dialysis bag and then placed in a closed container containing 50 ml PBS, acquiring the sink conditions, adjusted at 37 °C under gentle magnetic stirring. At predetermined time intervals, 1 ml of the medium was withdrawn and then replaced with an equal volume of fresh medium. The amount of RU released was calculated by HPLC at *λ*_max_ 360 nm. The release of free drug was also performed similarly. The *in vitro* release experiments were done in triplicate for each formulation.

#### *2.2.7. In vitro* hemolytic activity

Erythrocytes separated from rat blood was used to evaluate the potential hemolytic activity of the prepared SAA-coated LHPNPs. Serial dilutions of each nanosuspension type were prepared to assess the biocompatibility of each SAA at different concentration levels in the blood. The hemolytic assay was conducted according to the method adopted by Mourtas et al. (2009) with few modifications. Briefly, blood was collected from white albino healthy rats into sterile tubes containing EDTA-K3, centrifuged at 4000 rpm for 15 min and the supernatant plasma was then discarded. The obtained erythrocytes sediment was subjected to wash four times with sterile normal saline, then an erythrocyte suspension at a concentration of 2%(v/v) was prepared dispersed in normal saline and stored under refrigeration at 2–8 °C for further testing. On the day of study, 100 µl of the diluted nanosuspension was added to 900 µl of the erythrocytes suspension. The blood–NPs mixtures were gently mixed and allowed to stand for 1 h at room temperature. After that, 4 ml of normal saline was added to each incubated mixture so that the final volume suspension was adjusted to 5 ml, and then allowed to centrifugation at 10,000 rpm for 15 min to precipitate the erythrocytes. The supernatants obtained were assayed for hemoglobin, if present, using a UV–Visible spectrophotometer (Model UV-1601 PC; Shimadzu, Kyoto, Japan) at *λ*_max_ 414 nm. The hemolysis index was then calculated using the following equation:

$$\text{Hemolysis}\;(\%)=\frac{\left({\text{OD}}_{\text{t}}-{\text{OD}}_{\text{nc}}\right)}{\left({\text{OD}}_{\text{pc}}-{\text{OD}}_{\text{nc}}\right)}\times 100$$

Where OD_t_, OD_nc_, and OD_pc_ are the optical densities of the test sample, the negative control and the positive control, respectively.

The negative and positive controls were prepared by incubating the erythrocytes suspension with normal saline or deionized water, respectively.

#### 2.2.8. Pharmacokinetic study

Twenty-four white male albino rats (average weight ≈ 250 g.) were divided into 4 groups each of six animals. The study protocol was reviewed and approved by the Experiments and Advanced Pharmaceutical Research Unit (EAPRU), Faculty of Pharmacy, Ain Shams University on the use of the animals. The treatment was performed as follows; group I received RU solution prepared in 10% DMSO while groups II, III and IV received RU-loaded Tween 80-LPHNPs, TPGS-LPHNPs, Solutol-LPHNPs, respectively. All formulae were injected into the tail vein of the rats using a 1 cm^3^-U100 insulin syringe equipped with 28 G needle at RU dose level of 5 mg/kg (Zhu, 1998). Samples of 0.5 ml blood were then withdrawn from the retro-orbital venous plexus puncture at different time intervals; 0.25, 0.5, 1, 2, 4, 6, 8, 12, 24 and 48 h. Samples were collected in sterile tubes containing EDTA-K3 and centrifuged at 4000 rpm for 15 min. The supernatant plasma was separated, transferred into Eppendorf, and stored at −20 °C until analyzed using liquid chromatography/mass spectrometry (LC/MS).

The plasma RU concentrations versus time data for different groups were analyzed by non-compartmental estimations using PKsolver, the add-in program for pharmacokinetic and pharmacodynamic data analysis in Microsoft Excel. Maximum plasma concentration (*C*_max_) and the time to reach *C*_max_ (*T*_max_) were detected from plasma drug charts. The areas under the curve from time zero to last sampling time (AUC_0−t_), the area under the curve from time zero to infinity (AUC_0−∞_), and those under the first moment curve (AUMC) were determined. Mean residence time (MRT) was computed by dividing AUMC by AUC. The relative bioavailability (Fr), defined as the ratio of AUC_0−∞_ of either TPGS or Solutol-LPHNPs to that of Tween 80-LPHNPs at the same doses administered, was also calculated.

#### 2.2.9. Biodistribution study

Aiming to study the effect of different coating agents on the distribution of LPHNPs, 54 Swiss healthy mice (average weight ≈ 25 g) were randomly divided into three groups administered the three loaded formulae under study (Tween 80-LPHNPs, TPGS-LPHNPs and Solutol-LPHNPs) intravenously through the tail vein via a 28 G-needle insulin syringe at a dose of 5 mg/kg. At different time intervals after drug injection; 0.25, 0.5, 1, 2, 4 and 6 h, three mice from each group were sacrificed by decapitation. Different organs; brain, liver, spleen, and kidney were dissected from each mouse, washed with normal saline, blotted with filter paper to remove excess fluid, weighed and then stored at −20 °C until analysis.

On the day of analysis, organ samples are allowed to thaw and then subjected to homogenization after the addition of a calculated volume of normal saline. The tissue homogenates were centrifuged at 4000 rpm for 15 min, and the supernatants were collected for drug extraction. The RU concentrations in tissue samples were analyzed by LC/MS, similarly to plasma samples, and the mean RU amounts per g organ were then calculated and plotted versus time, and all pharmacokinetic parameters were computed as described earlier.

#### 2.2.10. Quantitative determination of RU using reversed phase high-performance liquid chromatography (RP-HPLC)

An isocratic RP-HPLC was adopted to quantify RU in all *in vitro* samples according to Kuntić et al. (2007) using Agilent Technologies (Santa Clara, CA, USA) 1200 series LC equipped with G 1311 A solvent delivery pump and G1315D diode array detector. A Kromasil^®^ C18 reverse-phase analytical column (5 μm particle size; 250 × 4.6 mm ID) was used and maintained at temperature =40 °C. The mobile phase constituted of a binary mixture of methanol–water 1:1 (v/v), pH 2.8 (adjusted with phosphoric acid) adjusted at flow rate of 1 mL/min. The wavelength of UV detector was set at *λ*_max_ 360 nm. The method was first validated according to the International Conference on Harmonization guidelines (ICH, Topic Q2A, Validation of Analytical Procedures: Methodology, PMP/ICH/281/95). The data was analyzed using ChemStation B.04.01 software (Santa Clara, CA, USA).

#### 2.2.11. Quantitative determination of RU in plasma using LC–MS/MS method

A sensitive, selective and accurate LC–MS/MS method was developed and validated for the determination of RU concentrations in plasma. Stock solution of hydrochlorothiazide internal standard (IS) was prepared by dissolving 10 mg in methanol and serially diluted with mobile phase to give a final working concentration of 200 ng/ml. A shimadzu Prominence (Shimadzu, Japan) series LC system equipped with degasser (DGU-20A3), solvent delivery unit (LC-20AB) along with auto-sampler (SIL-20 AC) was used to inject 20 µl aliquots of the processed samples on a Luna C (phenomenex, Torrance, CA, USA) (50 × 4.6) mm, 5 μm particle size. The isocratic mobile phase (pH 4.5) consisted of acetonitrile and (0.02 M) ammonium acetate buffer (70%, 30%, v/v) and 0.1% formic acid which was delivered at a flow rate of 0.50 ml/min into the mass spectrometer’s electrospray ionization chamber. All analysis was carried out at room temperature. Quantitation was achieved by MS/MS detection in negative ion mode for both RU and IS, using a MDS Sciex (Foster City, CA, USA) API-3200 mass spectrometer, equipped with a Turbo ionspray interface at 400 °C. The ion spray voltage was set at −4500 V. The nebulizer gas was set at 30 psi, curtain gas at 15 psi, auxiliary gas at 55 psi and collision gas at 9 psi. The compound parameters, namely, declustering potential, collision energy, entrance potential and collision exit potential were −1 V, −52 V, −10 V, and 19 V for RU and −45 V, −22 V, −10 V, and −12 V for hydrochlorothiazide (IS), respectively. The ions were detected in the multiple reaction monitoring mode, monitoring the transition of the *m/z* 608.9 precursor ion to the *m/z* 300.0 for RU and *m/z* 295.6 precursor ion to the *m/z* 268.9 for IS. Quadrupoles Q1 and Q3 were set on unit resolution and the analytical data were processed using Analyst software (Version 1.4.2).

#### 2.2.12. Statistical analysis

Each *in vitro* experiment was performed in triplicates; the average data and their standard deviations (SD) were then calculated. Results of the *in vivo* studies were expressed as mean ± standard error of the mean (SE). For statistical comparisons, a paired *t*-test was used; *p* < 0.01 was considered significant statistically.

The statistical relationships between the CPP and CQA in the experimental design were adopted using Design Expert^®^ version 7.0.0 software (Stat-Ease Inc., Minneapolis, MN, USA) applying design of experiment (DoE) by means of RSM and two-level full factorial design. The following polynomial equation model was employed fitting the experimental data and predicting the new trials (out of the design): *Y* = *β*_0_ + *β*_1_*X*_1_ + *β*_2_*X*_2_ + *β*_3_*X*_3_ + *β*_12_*X*_1_*X*_2_ + *β*_13_*X*_1_*X*_3_ + *β*_23_*X*_2_*X*_3_ + € where *Y* represents the predicted response, *X*_1_, *X*_2_, and *X*_3_ are the independent variables, *β*_0_ is the intercept, *β*_1_, *β*_2_, and *β*_3_ are the main effect coefficients, while *β*_12_, *β*_13_, and *β*_23_ are the two-way interaction coefficients, and €corresponds to the model residual. The quality of fit the experimental data by the polynomial model equation was expressed by the coefficient of determination (*R*^2^) and its adjusted *R*^2^. The analysis of variance (ANOVA) was adopted. The significance of each coefficient term and the lack of fit of the suggested model were evaluated *via p*-value and *F*-value with 95% confidence level. Main effects, contour and 3D-response surfaces plots were all designed.

#### 2.2.13. Statistical multi-objective optimization

After statistical analysis of the experimental design data, a numerical optimization was statistically adopted by applying the desirability function (D) which transforms the values of a response into [0,1] where 0 stands for a non-acceptable value of the response and 1 for ideal target values of this response (Derringer, 1994). With the same concept, this function was implemented for optimizing multiple responses simultaneously. A quality target product profile (QTPP) was identified aiming to optimize the CPP of the prepared formulations. The optimization was also illustrated graphically by contour, and 3-D response surface plots showing the desirability fractions and target areas. Aiming to compare the actual CQA of the optimized formulation with the predicted ones, the percentage bias (or prediction error) was computed from the following equation:

$$\%\text{Bias}=\frac{\left|\text{Predicted}-\text{Experimental}\right|}{\text{Experimental}}\times 100$$

## 3. Results and discussion

### 3.1. Compound isolation and structural elucidation

Phytochemical investigation of *C. officinalis* L. flowers resulted in the isolation of quercetin-3-*O*-rutinoside, the structure is shown in Figure S1. The isolated compound gave a green color upon spraying with FeCl_3_ reagent. The identification was performed on the basis of UV, NMR spectroscopy, and compared with literature data (Fathiazada et al., 2006). In addition, the isolated RU showed the same retention time (2.882 min.) on HPLC as the authentic RU (Sigma-Aldrich, St. Louis, IL, USA) (2.886 min), the HPLC chromatographic conditions and procedure were adopted according to Mostafa (2017). The isolated RU also showed a retention factor (*R*_f_) of 0.5 on TLC using the solvent system butanol:acetic acid:water (4:1:5, upper layer) which was in accordance with the authentic compound *R*_f_ value.

#### Quercetin-3-O-rutinoside (RU)

Yellow amorphous powder (100 mg), UV *λ*_max_ (nm) MeOH: 257, 266 (sh), 301 (sh), 362. ^1 ^H-NMR (400 MHz, DMSO) δ ppm: 1.00 (3 H, *d*, *J*= 4.5, CH_3_-6′′′), 3.10-3.72 (*m*, rutinosyl protons), 5.11 (1 H, *d*, *J*= 1.9, H-1′′′), 5.35 (1 H, *d*, *J*= 7.6, H-1′′), 6.20 (1 H, *d, J*= 2.5, H-6), 6.41 (1 H, *d, J*= 2.5, H-8), 6.85 (1 H, *d, J*= 8.3, H-5′), 7.55 (1 H, *d, J*= 2.2, H-2′), 7.59 (1 H, *d, J*= 8.3, 2.2, H-6′), 9.20 (*brs*, OH-3′), 9.68 (*brs*, OH-4′), 10.85 (*brs*, OH-7), 12.59 (*s,* OH-5). ^13 ^C-NMR (100 MHz, DMSO) δ ppm: 18.19 (C-6′′′), 68.70 (C-5′′′), 67.45 (C-6′′), 70.45 (C-2′′′), 70.83 (C-3′′′), 71.01 (C-4′′′), 72.30 (C-4′′), 74.52 (C-2′′), 76.35 (C-5′′), 77.30 (C-3′′), 94.05 (C-8), 99.14 (C-6), 101.19 (C-1′′), 101.60 (C-1′′′), 104.42 (C-10), 115.68 (C-2′), 116.72 (C-5′), 121.63 (C-6′), 122.05 (C-1′), 133.74 (C-3), 145.19 (C-3′), 148.85 (C-4′), 156.87 (C-2), 157.07 (C-5), 161.66 (C-9), 164.51 (C-7), 177.80 (C-4), as illustrated in Figure S2.

### 3.2. Preparation of LPHNPs loaded with RU

RU-loaded LPHNPs were successfully prepared using single-step modified nanoprecipitation technique where the lipid was dissolved in the water miscible organic solvent (acetone), not dispersed in water as usual, together with the polymer and drug. It could be assumed that the precipitation of PLGA into NPs simultaneously occurred with self-assembling of lecithin around them owing to hydrophobic interactions. The lipid hydrophobic tails were anchored to the polymeric hydrophobic core while the hydrophilic head was extended to the external aqueous medium stabilizing the hybrid NPs formed. The steric stabilization of LPHNPs was conventionally afforded by the PEG chains of a lipid-PEG element. Nevertheless, this was compensated in this study by; (1) higher *W*_lecithin_/*W*_PLGA_ ratios, as previously reported in such cases (Zheng et al., 2010) and (2) PEG-based SAA (Tween 80). Both approaches were anticipated to preserve the colloidal stability of the nanosuspensions obtained. In addition, the self-assembled hydrophilic outer shell of Tween 80 on the lipid–polymer surface adds to disguise the core hydrophobicity warranting the stealth property to particles after intravenous administration. The temperature of the nano-dispersions was maintained at 65 °C during preparation with the aim to conserve the homogenous dispersion of the lipid and the efficient rapid evaporation of the organic solvent as well. The drug to polymer ratio was maintained at 1:20 as at higher ratios (1:1, 1:5, 1:10, and 1:15), the prepared nanosuspensions suffered from conspicuous drug precipitation upon organic solvent evaporation and storage.

The effect of different PLGA amounts, *W*_lecithin_/*W*_PLGA_ ratios and Tween 80 concentrations was studied on drug EE, PS, and PDI. The formulation results, depicted in Table 1, show that LPHNPs were characterized by EE, PS, and PDI ranging from 31.35% to 83.15%, 147.60 to 333.85 nm and 0.153 to 0.424, respectively.

### 3.3. Statistical analysis and modeling

A DoE was conducted to evaluate and quantify the effect of independent factors on the selected CQA. A 2^3^-full factorial design was applied and the product design space was defined leading to eight sets of experiments with three replicates. The statistical significance of three CPP, i.e. polymer amount, *W*_lecithin_/*W*_PLGA_ ratio and Tween 80 concentration, each at two levels, on different LPHNPs CQA; EE, PS, and PDI; were considered using RSM.

The Box–Cox Plot for power transforms was initially diagnosed. This is typically used as a guideline for selecting the correct power law transformation (lambda) set at the minimum point of the curve generated by the natural log of the sum of squares of the residuals (Hashad et al., 2016). Aiming to attain the best fitting models, a transformation was only recommended for PS response (figures not shown) where the suggested power corresponding to the best lambda was – 1.37, as depicted in Table S1.

The statistical models were developed by regression analysis using the experimental data for the drug EE, PS, and PDI, as displayed in Table S1. The regression models were found significant at *p* < .0001. The equation models were suggested as two-factor interaction (2FI) for both EE and PS while the linear equation was recommended for PDI. The coefficient of determination (*R*^2^) was calculated to be higher than 0.9 for all responses under study (Table S1) indicating the satisfactory adjustment of the suggested models to the experimental data as well as the high predictability of these models. The non-significant ‘Lack of Fit’ (*p* > 0.05) for all models is considered a good sign implying its insignificance relative to the pure error. The ‘Predicted *R*^2^’ were in reasonable agreement with the ‘Adjusted *R*^2^’ for all models under study. The ‘Adequate Precision’ measures the signal to noise ratio. A ratio greater than 4 is desirable indicating an adequate signal, hence all models can be used to navigate the design space.

By means of observation of ANOVA analysis, based on Table S2, it was found that all main effects model terms (*X*_1_, *X*_2_, and *X*_3_) were found significant in terms of EE and PS (*p* < 0.05). In the case of PDI, only *X*_1_ was considered significant. The interaction model terms were found significant in EE and PS models only. Note that the non-significant model terms were omitted, so that the model reduction may improve its applicability.

**Figure 1.**
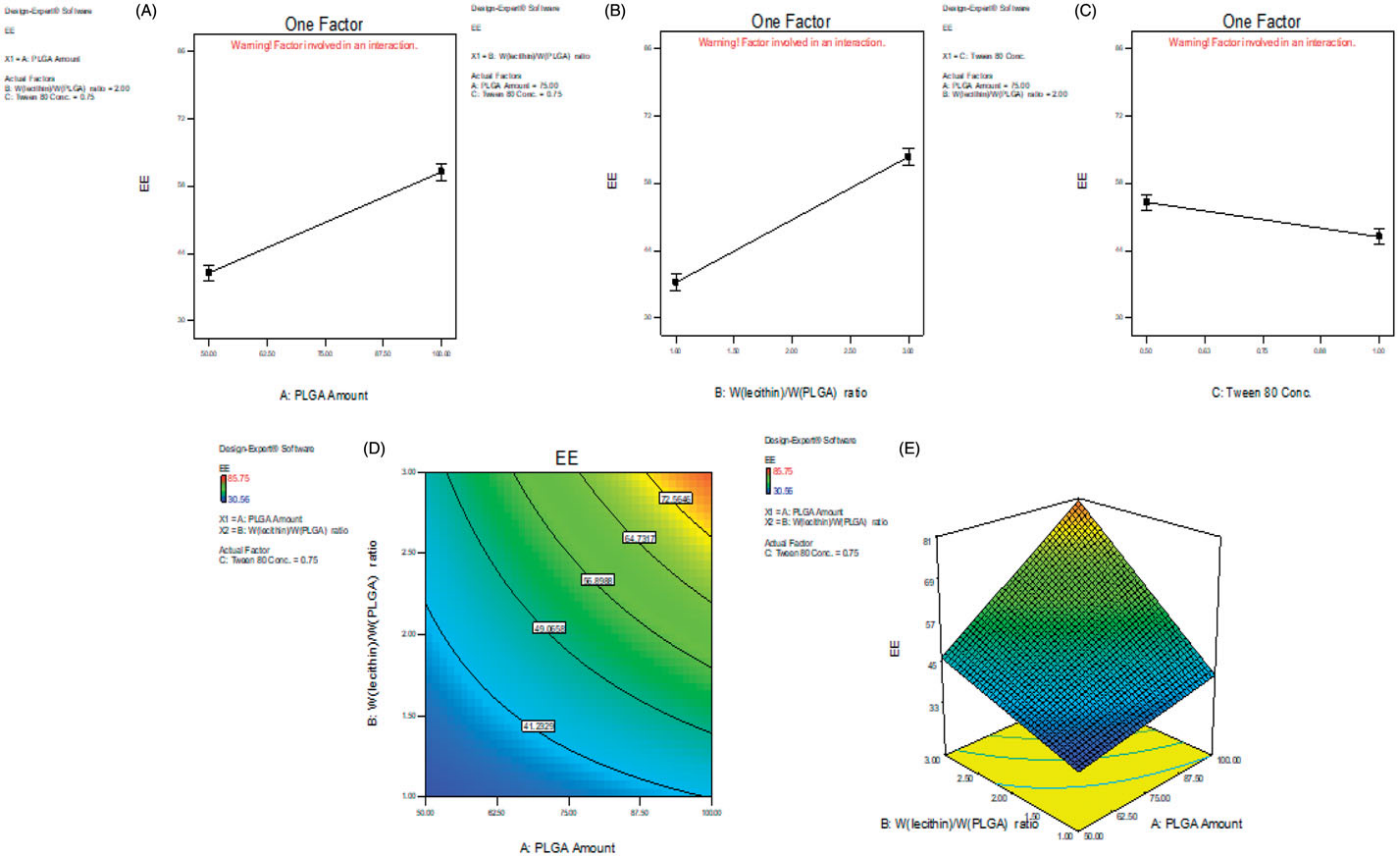
Main effect plots (A–C) illustrating the effect of each CPP on EE. Contour and 3D-surface plots showing (D, E) the interaction effects of PLGA amount (*X*_1_) and *W*_lecithin_/*W*_PLGA_ ratio (*X*_2_) on EE.

The ANOVA results (Table S2) show that all factors studied had significant effects on RU EE (*p < *.01), being the effect of *W*_lecithin_/*W*_PLGA_ ratio the highest. A clear positive influential EE response was associated with PLGA amount and *W*_lecithin_/*W*_PLGA_ ratio and, whereas the SAA concentration impacted a negative effect, as presented in Figure 1(A–C), respectively, and revealed in Table S1 equations. As illustrated in contour and 3D-response surface plots (Figure 1(D,E), respectively), the same positive effect on EE attribute was observed when PLGA amount was combined with *W*_lecithin_/*W*_PLGA_ ratio parameter. The highest RU EE was attained at the highest levels (+1) of both factors; 100 mg PLGA and 3/1 *W*_lecithin_/*W*_PLGA_ ratio.

**Figure 2.**
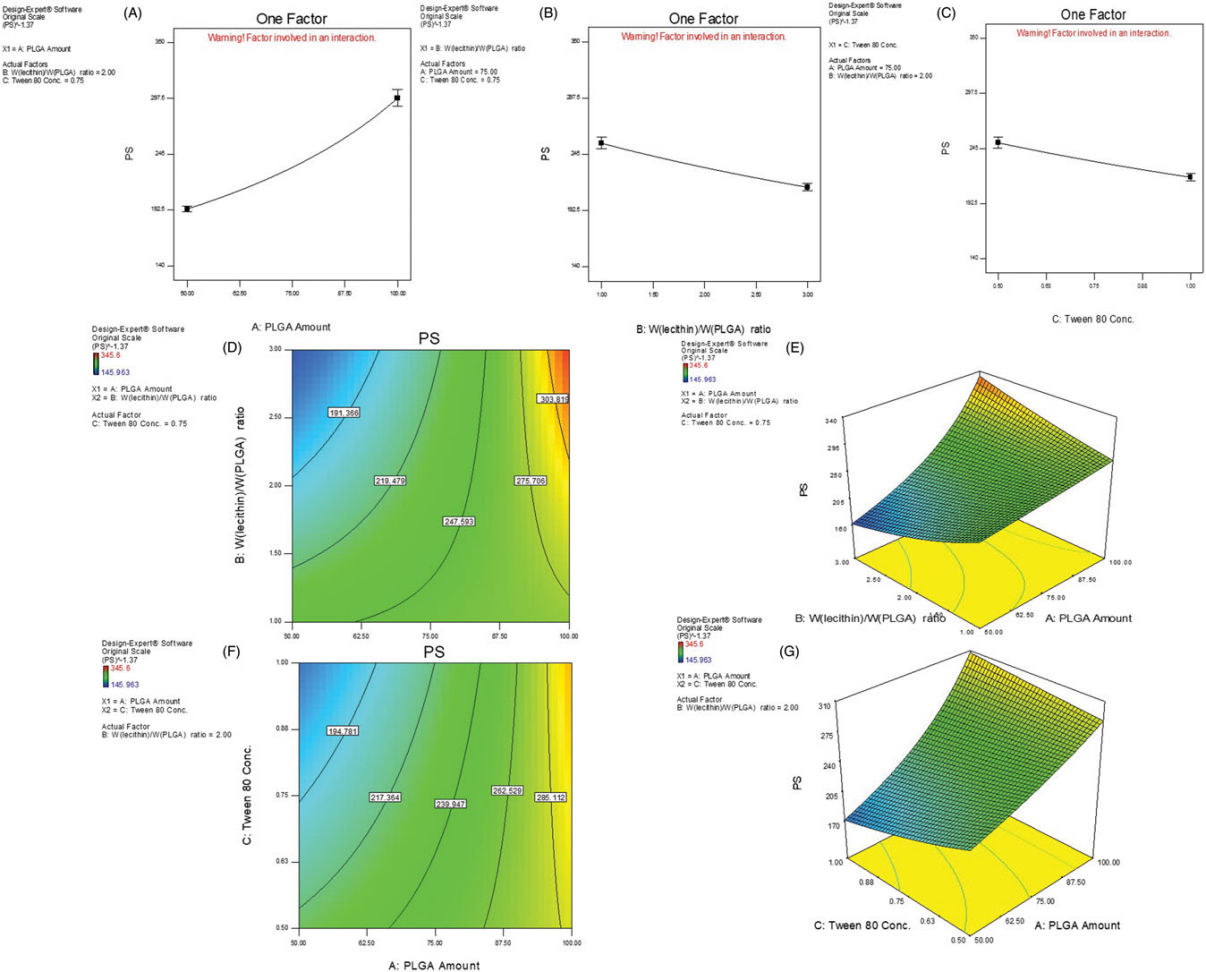
Main effect plots (A–C) illustrating the effect of each CPP on PS. Contour and 3D-surface plots showing (D, E) the interaction effects of PLGA amount (*X*_1_) and *W*_lecithin_/*W*_PLGA_ ratio (*X*_2_), and (F, G) that of PLGA amount (*X*_1_) and Tween 80 concentration on PS.

Table S2 also shows that NPs PS is significantly affected by all CPP under study (*p <* .01) with the most prominent effect goes for PLGA amount. Increasing polymer amount led to an enlargement in PS while the opposite followed in case of *W*_lecithin_/*W*_PLGA_ ratio as well as SAA concentration, as shown in Figure 2(A–C), respectively. The contour and 3D-response surface plots (Figure 2(D,E), respectively) illustrate a special interaction between *X*_1_ and *X*_2_ where at high level (+1) of *W*_lecithin_/*W*_PLGA_ ratio (3/1), the both lowest and highest PS were manifested yet when combined with the low (−1) and high (+1) levels of PLGA amount, respectively. Also, the lowest PS was reached when the lower level (−1) of PLGA amount was accompanied with the higher level (+1) of Tween 80 concentration, as revealed in Figure 2(F,G).

The effect of polymer amount in the prepared hybrid NPs was found similar to that in case of nonhybrid (polymeric) NPs. Increasing polymer content increased drug EE, PS and heterogeneity of the NPs formed. This could be ascribed to the rapid precipitation of the polymer on the surface of the internal droplets hindering drug diffusion, hence EE improved. The increased viscosity of the dispersed phase increases the diffusion resistance of drug molecules from the organic to the aqueous phase leading to the formation of non-uniform larger particles of longer diffusional pathways for the drug, thereby reducing its loss and increasing its entrapment (Kandel et al., 2011).

The increase in *W*_lecithin_/*W*_PLGA_ ratio, i.e. the rise in SPC concentration was linked with an improvement in drug EE and unexpectedly a reduction in mean NPs size. Similar to the effect of PLGA amount, the increased lipid concentration increases the viscosity of the internal phase in which it is dissolved, preventing the drug diffusion and leakage out from polymeric matrix to the external aqueous phase which in turn led to superior drug entrapment (Yang et al., 2000). It has been previously reported that increasing lecithin concentration beyond its critical micelle concentration leads to the formation of assembled vesicles adjacent to LPHNPs in the process medium which could, in turn, enhance RU EE (Zhang et al., 2008; Sailor & Park, 2012). Surprisingly, the *W*_lecithin_/*W*_PLGA_ ratio is inversely proportional with the mean PS of NPs formed. This might be attributed to the co-presence of small-sized liposomes in case of high lipid content as above mentioned which could decrease the overall measured size of formed NPs. Likewise, the lower *W*_lecithin_/*W*_PLGA_ ratio led to particle aggregation owing to the withdrawal of lipid stabilization effect in this case which, in turn, causes an increase in PS of hybrid NPs (Zhang et al., 2008).

The increase in Tween 80 concentration led to a significant decrease in drug EE and PS of LPHNPs. This decrease in RU EE could be attributed to the increase in drug diffusion and partitioning from internal droplets to the external aqueous environment which, in turn, leads to a reduction in PS particularly if some drug particles were deposited on NPs surface. Moreover, the greater SAA concentration is typically translated into a higher number of SAA molecules localized at the organic solvent–water interface which consecutively reduced the interfacial tension during the emulsification process and promoted the formation of smaller oil droplets.

### 3.4. Statistical optimization using the desirability function (D)

After statistical analysis, a numerical optimization was conducted aiming to select the most optimum CPP for the preparation of LPHNPs. The optimal quality target product profile (QTPP) was set in terms of EE, PS, and PDI, as tabulated in Table S3. Drug entrapment was fixed at maximum values while NPs size and size distribution were adjusted to a certain limit, i.e. ≤250 nm and ≤0.3, respectively, above which CQA are considered out of the target goals. Three-dimensional-response surface plots (Figure S3(A and B)) were constructed relating *X*_1_ and *X*_2_ at 0.5 and 1% Tween 80 concentration, respectively. These plots show the optimal goal parameters for attaining the QTPP required. As displayed in Figure S3(A and B), the highest D attained was 0.69 and 0.64, at 0.5% and 1% SAA concentration, respectively. The target areas in plots are manifested in yellowish green color. Therefore and based on D values, the optimal CPP; PLGA amount, *W*_lecithin_/*W*_PLGA_ ratio and Tween 80 concentration, were adjusted to 75 mg, 3/1 and 0.5%, respectively.

### 3.5. Determination of CQA of the optimized formulation

As illustrated in Figure S3(C–E), the predicted CQA of the optimized formula consisting of 75 mg PLGA, 3/1 *W*_lecithin_/*W*_PLGA_ ratio and 0.5% Tween 80 were 69.13%, 241.89 nm and 0.3 for EE, PS, and PDI, respectively. The optimized formulation was then prepared using the optimized CPP and all actual CQA were measured, recorded in Table S4 and compared with the predicted values. By computing the prediction error percent, it could be concluded the validity and predictability of the model equations generated from DoE owing to the small %bias which did not exceed 20% (Abdel-Messih et al., 2017).

### 3.6. Preparation of LPHNPs prepared with different PEG-based SAA

Using the optimized CPP, the chosen PEG-based SAA i.e. TPGS and Solutol were employed replacing Tween 80 in the preparation of LPHNPs. The CQA (EE, PS, and PDI) of the prepared NPs were also measured and tabulated in Table 2. All prepared NPs showed relatively comparable results. Indeed, TPGS-LPHNPs significantly demonstrated (*p* < .05) the highest EE and the lowest PS among all prepared NPs. The fact could be attributed to the apparent increase in viscosity of the aqueous phase in which SAA was dissolved, owing to the higher Mw of TPGS (1513 g/mol) compared with other SAA (Tween 80 *M*_w_ = 1310 g/mol and Solutol HS15 *M*_w_= 963.24 g/mol), which would reduce the drug diffusion and loss in the surrounding medium, and hence improve drug EE. In the same context, the efficiency of TPGS as emulsifier has been previously reported during the preparation of polymeric NPs (Mu & Feng, 2003). This might be accounted on its effective alignment at oil/water interface causing a substantial reduction in the free energy at the interface and thus resulting in lower PS.

**Table 2.** CQA data of the optimized LPH NPs prepared with different PEG-based SAA.

	RU EE (%) ±SD	PS (nm) ± SD	PDI ± SD	ZP (mV) ± SD
Tween 80-LPH NPs	64.32 ± 1.11	272.50 ± 3.39	0.272 ± 0.029	−5.03 ± 0.18
TPGS-LPH NPs	74.23 ± 2.14	203.00 ± 2.20	0.251 ± 0.022	−2.52 ± 0.52
Solutol HS 15-LPH NPs	68.06 ± 1.50	232.4 ± 4.01	0.339 ± 0.010	−1.76 ± 0.33

### 3.7. Recovery and purification of the prepared LPHNPs

A dialysis-based purification step was applied aiming to recover NPs as well as eliminate excess SAA and unentrapped drug from the prepared nanosuspensions. Only 30 min. was found sufficient to get rid of all free drug, after which the release of entrapped RU began. Though this short dialysis duration was found not satisfactory to wipe out completely the excess SAA present, this was confirmed by the non-significant alteration (*p* > .05) in PS and surface charge of the dialyzed suspensions (data not shown) in comparison with the non-dialyzed ones, as recorded in Table 2. Indeed, the almost neutral surface of LPHNPs reveals the high NP surface coverage with the nonionic SAA being studied particularly for their stealth effect and their role in enhancing brain delivery. Therefore, the level of SAA was maintained in NP formulations without purification aiming to conserve the stability of NPs formed and their potential toxic effects were studied by *in vitro* hemolysis assay.

### 3.8. Characterization of the prepared LPHNPs

#### 3.8.1. Differential scanning calorimeter

The DSC thermograms of RU, PLGA, SPC and loaded LPHNPs are illustrated in Figure 3(A). The drug shows two main endothermic peaks at 156 and 182°C indicating its melting points, as reported in the literature (Sri et al., 2007). These peaks are followed by successive peaks at 240, 258, and 264 °C. This could be attributed to the molecular rearrangement of RU polymorph due to the presence of sugars in its molecule, confirming its decomposition above 200 °C (Costa et al., 2002). The polymer reveals an endothermic peak at 51.72 °C owing to its glass transition temperature (*T*_g_) (Ishak et al., 2014) whereas SPC exhibits a gel–liquid crystalline phase transition at 43.28 °C followed by consecutive irregular peaks above 130 °C denoting its degradation at higher temperatures (Fathalla et al., 2015). The thermograms of all loaded NPs show the disappearance of all characteristics peaks of their components except those pertaining to PLGA and/or SPC. The disappearance of RU melting points confirms the molecular dispersion of the drug within the nanocarrier structures.

Figure 3.(A) DSC thermograms of RU, PLGA, SPC and loaded LPH NPs. (B) HR-TEM micrographs of RU-loaded Tween 80-LPH NPs (a), TPGS-LPH NPs (b), Solutol-LPH NPs (c) and polymeric NPs prepared with 50 mg PLGA and 1% Tween 80 (d). (C) *In vitro* release profiles of free RU and RU-loaded LPH NPs. (D) Percent Hemolysis induced by incubation of various types of RU-loaded LPH NPs with rat blood at different SAA concentrations. Each data represents the mean ± SD (*n* = 3). The black dotted line in (d) denotes the permissible threshold hemolysis percent. NS; Nonsignificant difference at *p* > .05.

**Figure:**
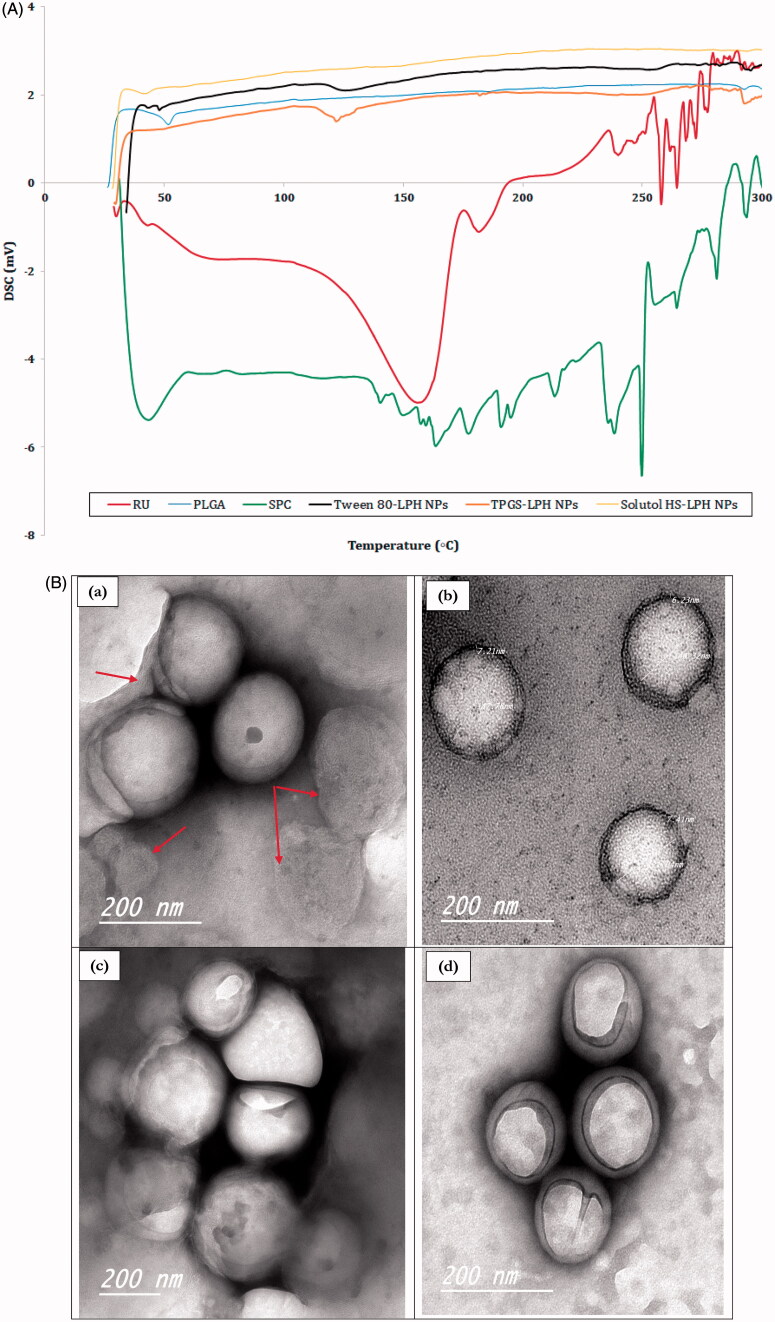

**Figure:**
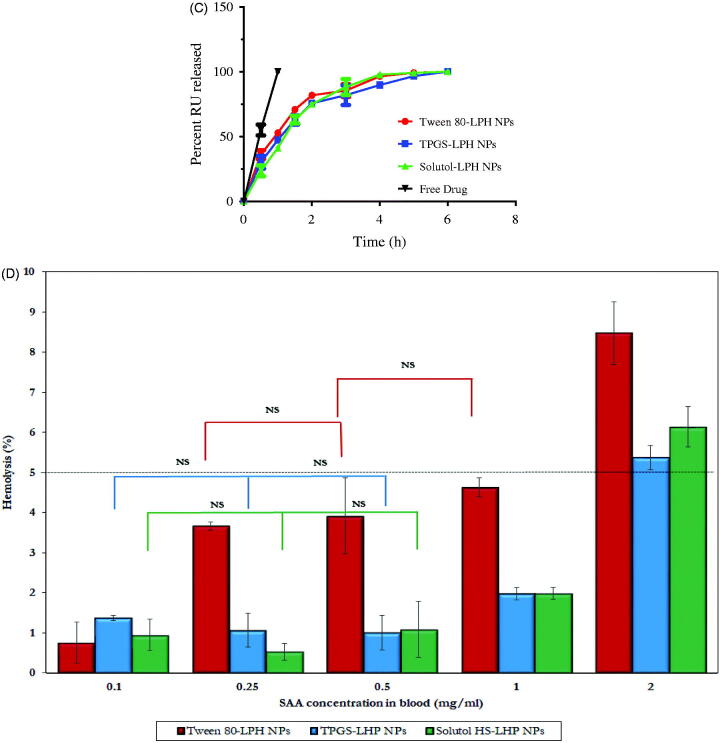

#### 3.8.2. Particle morphology using HR-TEM

TEM was used to visualize the morphology of RU-loaded LPHNPs coated with different PEG-based SAA. As illustrated in Figure 3(B (a–c)), all hybrid particles show spherical shape with an almost homogenous size distribution. The PS of NPs observed in TEM images are relatively smaller than that measured by DLS (Table 2). This could be explained by the fact that samples for TEM imaging were measured in the dry state while DLS technique measures the hydrodynamic particle diameters. The core–shell structure of the particles is clearly recognized by the difference in contrast between the two regions where the lipid shell attains roughly 7 nm thickness, as depicted in Figure 3(B(b)). The multi-lamellar lipid shells are detected around polymeric cores, further few discrete phospholipid vesicles *co*-exist adjacent to hybrid particles, as pointed out by red arrows in Figure 3(B(a)). This could be due to the high SPC concentration incorporated, the excess lipid is assumed to be self-organized in multi-layered deposits onto PLGA cores or into vesicle-like structures (Yi et al., 2014). The outermost SAA coats are greatly perceived folded up forming a cover that shields the majority of particles. Besides, the image of polymeric NPs was captured for comparison; showing similar SAA shield without the appearance of any lipidic shells (Figure 3(B(d))).

### *3.9. In vitro* drug release study

The *in vitro* release study of RU from different formulations was performed using dialysis bag technique in PBS pH 7.4 adjusted at 37 °C under sink conditions. The comparative drug release profiles of different types of loaded LPHNPs were illustrated in Figure 3(C). A control experiment was conducted for free drug showing a complete RU release after 1 h. As observed in Figure 3(C), all NP formulations under study showed gradual drug release over 6 h allowing a sustained effect. Despite the difference in shell materials, the RU release profiles are considered analogous. The sustainment in RU release from LPHNPs seemed unexpectedly restricted. The fact might be explained based on the glycosidic chemical structure of RU where it carries many hydroxyl (–OH) polar groups even though its water insolubility (0.013 g/100 ml) (Krewson & Naghski, 1952). Hence, it was assumed that the drug is localized onto the surface of the hydrophobic PLGA core not within the polymeric matrix, being attached to the polar parts of the amphiphilic lipid coat by hydrogen bonds/hydrophobic interactions (Ahmad et al*.,* 2016). This might guarantee a short diffusion pathway for the release of drug elucidating the short duration of sustainment observed among the release profiles of LPHNPs. Furthermore, the presence of considerable amount of SAA in each formula could add to this effect by enhancing drug release (Guan et al., 2016).

### *3.10. In vitro* hemolytic activity

As the prepared RU-loaded nanosuspensions are intended to be administered by IV route, thus they have to be tested for hemolytic toxicity studies. The hemolysis percent induced by various types of LPHNPs were measured and their data are illustrated in Figure 3(D). It is obvious the significant higher hemolytic activity of Tween 80-based NPs (*p* < .05) compared with the corresponding nanocarriers either coated with TPGS or Solutol at all SAA concentration levels. The low toxicities of TPGS and Solutol were previously reported by Pooja et al. (2014) and Yan et al. (2016). The percent hemolysis increased as a function of increasing SAA concentration from 0.1 to 2 mg/ml in blood. A nonsignificant difference (*p* > .05) in hemolysis was detected in case of Tween 80 between concentrations 0.25 and 0.5 mg/ml, 0.5 and 1 mg/ml while for TPGS and Solutol the insignificance was recognized at concentration range 0.1–0.5 mg/ml. Based on numerous studies, the *in vitro* percent hemolysis is assessed as ‘no concern’ when it ranges from 5% to 25% (Dobrovolskaia et al., 2008). However, the permissible threshold is limited to 5% according to the new consensus ASTM E2524-08 -Standard test method for analysis of hemolytic properties of nanoparticles (ASTM International, West Conshohocken, PA, USA 2000). It was observed that the hemolysis percent exceeded 5% in all NP types when SAA concentration attained 2 mg/ml. These results confirmed the biocompatibility of all LPHNPs at SAA levels lower than 2 mg/ml in blood, hence such concentration limit was maintained in all subsequent *in vivo* studies.

### 3.11. Pharmacokinetic assessment of RU-loaded LPHNPs

**Figure 4.**
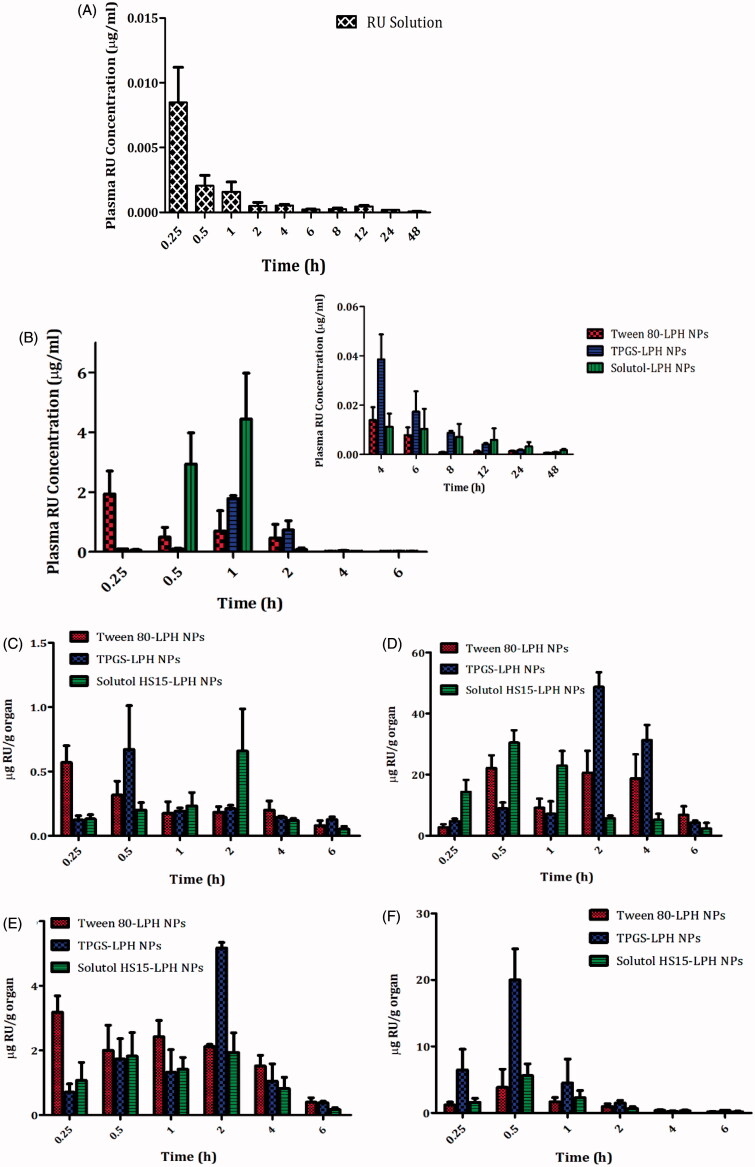
Plasma RU concentration *versus* time after IV Administration of the drug solution (A) and loaded LPH NPs (B) to rats at dose 5 mg/kg. The insert in (B) shows the plasma RU concentration *versus* time at time intervals ranging from 4 to 48 h. Each point represents mean ± SE (*n* = 6). Tissue distributions of RU in brain (C), liver (D), spleen (E), and kidney (F) after IV administration of different loaded LPH NPs to mice at dose 5 mg/kg. Each point represents mean ± SE (*n* = 3).

The pharmacokinetic study of RU-loaded LPHNPs based on different PEG-SAA types as well as RU solution was explored by tail vein injection to rats at 5 mg/kg dose. The plasma drug concentration versus time charts are plotted in Figure 4, and the pharmacokinetic parameters are collected in Table 3. It is obvious the extremely low RU concentration in plasma after solution injection, as illustrated in Figure 4(A). The fact could be attributed to the extensive hepatic metabolism of RU after intravenous administration. This finding was recently reported by Choi et al. (2016) where the authors demonstrated that large amount of intravenously injected ^125^I-radiolabeled RU was metabolized in liver, and most of it was transferred to the small intestine via the bile within 1 h only.

**Table 3.** Plasma pharmacokinetics parameters of RU after IV administration of drug solution and different types of LPH NPs to rats at 5 mg/kg dose.

	Data (mean^a^ ±SE)						
	*T*_max_ (h)	*C*_max_ (µg/ml)	AUC_0–t_ (µg/ml*h)	AUC_0_–_∞_ (µg/ml*h)	AUMC_0–∞_ (µg/ml*h^2^)	MRT_0–∞_ (h)	Fr
RU solution	0.33 ± 0.07	0.0088 ± 0.0025	0.0258 ± 0.0066	0.0278 ± 0.0066	0.2069 ± 0.0567	7.76 ± 2.01	–
Tween-LPH NPs	0.33 ± 0.08	1.91 ± 0.39	4.45 ± 2.33	4.46 ± 2.34	4.60 ± 2.86	1.90 ± 1.17	–
TPGS-LPH NPs	1.00 ± 0.00*	1.78 ± 0.10^NS^	2.72 ± 0.42*	2.72 ± 0.42*	5.75 ± 0.81^NS^	2.13 ± 0.06^NS^	0.61
Solutol-LPH NPs	0.67 ± 0.17*	4.40 ± 1.31*	4.39 ± 2.56^NS^	4.41 ± 2.57^NS^	8.82 ± 4.81^NS^	3.04 ± 0.72^NS^	0.99

^a^ Average of six determinations.

*Significant difference at *p* < .05 compared with Tween-LPH NPs.

NS: nonsignificant difference at *p* > .05 compared with Tween-LPH NPs.

SE: standard error of the mean; *T*_max_: time to maximum drug concentration; *C*_max_: maximum plasma drug concentration; AUC: area under the concentration vs time curve; AUMC: area under the first moment curve; MRT: mean residence time; Fr: relative bioavailability = ratio of AUCs (TPGS or Solutol-LPH NPs to Tween-LPH NPs).

Although the short residence duration of RU in case of all loaded NPs attaining a MRT of 1.90, 2.13, and 3.04 h, a significant improve in RU bioavailability (*p* < .05) by about 160-fold, 98-fold, and 159-fold was perceived for Tween, TPGS, and Solutol-based particles, respectively, relative to the drug solution (Table 3). This could be attributed to the stealth effect triggered by the PEG moieties in SAA structures coating NPs, which affords a little recognition by macrophages and hence a relatively long-circulation property to NPs (Sheng et al., 2009). Besides, the higher drug bioavailability associated with LPHNPs might be counted on the ultimate interactions of RU with phospholipids (Ahmad et al., 2016), as described earlier in *in vitro* release studies, which would control the drug release, hence impeding its fast uptake and metabolism by liver cells, and improving its bioavailability.

The higher peak plasma levels were attained by LPHNPs sheathed with Solutol, followed by Tween, and then TPGS (Table 3). The *C*_max_ of Solutol-LPHNPs was higher by about 2.3 folds than that of Tween-coated particles. Nevertheless, both Tween and Solutol-based formulations were found to increase the overall systemic availability of RU where they showed equivalent bioavailability expressed by Fr ≈ 1 (Table 3). Surprisingly, TPGS-LPHNPs exhibited a much lower bioavailability compared with former NPs, this was confirmed by lower AUCs, and Fr, as manifested in Table 3. Hence, the *in vivo* fate of the NPs should be investigated through the biodistribution studies.

### 3.12. Biodistribution study

The distributions of different LPHNPs in various organs; brain, liver, spleen, and kidney, for a 6-h period post-administration intravenously to mice are illustrated in Figure 4(C–F), and the pharmacokinetic parameters are recorded in Table 4.

**Table 4.** Pharmacokinetics parameters of tissue distribution of RU after IV administration of Tween-LPH NPs, TPGS-LPH NPs and Solutol-LPH NPs to mice at 5 mg/kg dose.

	Brain (mean^a^ ±SE)			Liver (mean^a^ ±SE)			Spleen (mean^a^ ±SE)			Kidney (mean^a^ ±SE)		
	Tween-LPHNPs	TPGS-LPHNPs	Solutol-LPHNPs	Tween-LPHNPs	TPGS-LPHNPs	Solutol-LPHNPs	Tween-LPHNPs	TPGS-LPHNPs	Solutol-LPHNPs	Tween-LPHNPs	TPGS-LPHNPs	Solutol-LPHNPs
*T*_max_ (h)	0.25 ± 0.00	1.17 ± 0.42^b^	1.17 ± 0.44^b^	1.00 ± 0.50	2.00 ± 0.00^b^	0.67 ± 0.17^NS^	0.58 ± 0.22	2.00 ± 0.00^b^	0.92 ± 0.55^NS^	1.17 ± 0.44	0.50 ± 0.00^b^	0.67 ± 0.17^NS^
*C*_max_ (µg/g)	0.57 ± 0.13	0.67 ± 0.34^NS^	0.66 ± 0.33^NS^	24.92 ± 5.20	48.74 ± 4.81^b^	33.67 ± 1.08^b^	3.67 ± 0.26	5.18 ± 0.17^b^	2.77 ± 0.30^b^	4.15 ± 2.60	20.02 ± 4.68^b^	6.39 ± 1.02^NS^
AUC_0–t_ (µg/g^b^ h)	1.14 ± 0.27	1.11 ± 0.31^NS^	1.31 ± 0.53^NS^	71.16 ± 8.58	149.93 ± 18.00^b^	53.48 ± 3.61^b^	10.00 ± 0.63	12.08 ± 1.14^b^	6.76 ± 0.33^b^	5.43 ± 1.37	15.69 ± 4.02^b^	6.10 ± 0.58^NS^
AUC_0–∞_ (µg/g^b^ h)	1.59 ± 0.56	1.80 ± 0.41^NS^	1.50 ± 0.47^NS^	79.54 ± 9.39	157.08 ± 18.72^b^	60.89 ± 8.85^b^	11.14 ± 1.20	12.69 ± 1.19^NS^	7.17 ± 0.53^b^	5.83 ± 1.25	16.69 ± 3.97^b^	6.53 ± 0.41^NS^
AUMC_0–∞_ (µg/g^b^ h^2^)	8.33 ± 4.68	11.39 ± 2.84^NS^	4.86 ± 1.24^NS^	235.80 ± 75.35	484.54 ± 59.11^b^	165.31 ± 66.32^NS^	33.63 ± 8.81	32.26 ± 4.69^NS^	18.44 ± 3.78^b^	12.42 ± 1.57	25.72 ± 5.35^b^	12.16 ± 0.53^NS^
MRT_0–∞_ (h)	4.41 ± 1.18	6.26 ± 4.25^NS^	3.52 ± 0.78^NS^	2.93 ± 0.75	3.08 ± 0.03^NS^	2.53 ± 0.65^NS^	2.93 ± 0.43	2.52 ± 0.15^NS^	2.53 ± 0.33^NS^	2.31 ± 0.47	1.58 ± 0.20^b^	1.89 ± 0.19^NS^
Fr	–	1.13	0.94	–	2.26	0.77	–	1.14	0.64	–	2.69	1.12

^a^ Average of 3 determinations.

^b^ Significant difference at *p* < 0.05 compared with Tween-LPH NPs.

^NS^ Nonsignificant difference at *p* > 0.05 compared with Tween-LPH NPs.

SE: Standard error of the mean; *T*_max_: Time to maximum drug concentration; *C*_max_: Maximum plasma drug concentration; AUC: Area Under the concentration vs time Curve; AUMC: Area Under the first Moment Curve; MRT: Mean Residence Time; Fr: Relative Bioavailability = Ratio of AUCs (TPGS or Solutol-LPH NPs to Tween-LPH NPs).

Interestingly, NP surface treatment with either TPGS or Solutol exhibited comparable uptake by brain cells relative to the gold standard (Tween 80), expressed by non-significant differences in AUCs (*p* > .05) and Fr values almost equal 1 **(**Figure 4(C) and Table 4). The utilization of these SAA in overcoming BBB obstacle and enhancing drug accumulation within brain cells has been reported in previous studies based on different underlying mechanisms. Tween 80-coated particles were testified to adsorb apolipoproteins E and/or B in blood, rendering particles similar to plasma lipoproteins, hence capable to be up taken by brain endothelial cells via receptor-mediated endocytosis (Göppert & Müller, 2003; Koziara et al., 2003). TPGS was described to enhance drug uptake into brain cells via a nonspecific absorption by the adsorptive-mediated endocytic pathway (Zhang et al., 2012). Additionally, TPGS has been reported to inhibit P-gp receptors predominately expressed on epithelia apical membrane in brain endothelial cells, restraining the drug efflux transport through the inhibition of P-gp ATPase (Zastre et al., 2008; Collnot et al., 2010). Solutol shares with TPGS the same P-gp inhibition mechanism, as reported by Hoosain et al. (2015). Lately, Solutol was confirmed to cross BBB via the same pathway executed by Tween 80 (Kasongo et al., 2011). The latter mechanism is preferentially supported by the results of this study.

As observed in Table 4 and Figure 4(D–F), the tissue distributions of RU in RES organs (liver, spleen, and kidney) from different LPHNPs noticeably vary. Solutol-LPHNPs exhibited the least significant drug accumulation (*p* < .05) especially in liver and spleen, expressed by lower AUCs. This excitingly reveals the efficiency of this PEG-SAA in sheathing NPs away from macrophages recognition, compared with other SAAs. Unexpectedly, TPGS-LPHNPs exhibited higher drug accumulation in RES organs; liver > kidney > spleen, reflected by significant higher AUCs (*p* < .05) compared with Tween-sheathed particles, translated by high Fr values, computed as 2.26, 2.69 and 1.14 in liver, kidney and spleen, respectively. The predominant cause of NPs loss from the systemic circulation is its phagocytic uptake by RES. These results demonstrate that the surface modification with TPGS did not avoid the opsonization process and engulfment of particles by macrophages. Similar finding was previously reported by Durán-Lobato et al. (2014). There is an inverse correlation between the increased NP retention in blood and its accumulation in RES organs (liver, kidney, and spleen). That’s to say the more NPs captured by macrophages, the lower their circulation in blood, and hence the reduced drug bioavailability. This was exactly what occurred in case of TPGS-NPs which showed the lowest RU availability in plasma confirming the sequestration of particles by RES organs. Moreover, RU is well-reported as a substrate for P-gp receptors highly expressed in liver and kidney tissues and responsible for the efflux of drugs out of cells (Zhang et al., 2013). Hence, the presence of TPGS, which could specifically inhibit P-gp receptors (Collnot et al., 2010), might be the reason for the increase in drug concentrations within such organs.

## 4. Conclusions

We have successfully developed the novel LPHNPs composed of a PLGA polymeric core and soybean lecithin intertwined, for the first time, with a PEG-SAA instead of PEG-lipid, forming the lipid shell for the effective delivery of RU to the brain. Single-step modified nanoprecipitation method was adopted to prepare LPHNPs using Tween 80. A 2^3^-full factorial design was applied to study the effect of different CPP, and their statistical significance on CQA of NPs formed using RSM. The prepared LPHNPs were optimized using the desirability function at 75 mg PLGA amount, 3/1 *W*_lecithin_/*W*_PLGA_ ratio, and 0.5% Tween 80 concentration, characterized by 64.32%, 272.50 nm, and 0.272 for EE, PS, and PDI, respectively. The optimized CPP were employed for replacing Tween 80 with other PEG-SAA, TPGS, and Solutol, which produced comparable CQA. The molecular dispersion of RU within the nanocarrier matrices were confirmed by DSC. All hybrid particles showed spherical shape in TEM images with multi-lamellar phospholipid shells and SAA shields. The drug release profiles seemed analogous irrespective to the type of NPs, which extended for 6 h. The biocompatibility of the prepared NPs was confirmed by a hemolytic test. The pharmacokinetic study revealed a significant improvement in RU bioavailability (*p* < .05) for all prepared hybrid NPs relative to the drug solution. However, TPGS–LPHNPs exhibited a much lower drug bioavailability compared with the other NPs. The biodistributions of different LPHNPs were also studied. Nonsignificant differences in brain drug uptake (*p* > .05) were detected among the different types of LPHNPs However, NP surface treatment with TPGS specifically exhibited a noticeable higher phagocytic uptake in RES organs. The results of the present study highlighted the potential applicability of Solutol and TPGS in brain targeting being equivalent to the gold standard Tween 80. This study opens the door for further investigations in the field of neuro-pharmaceuticals aiming to confirm the competency of these PEG-SAA in brain delivery, phagocytic uptake evasion, and long circulation in blood.

## Supplementary Material

IDRD_Ishak_et_al_Supplemental_Content.docx
