# Supplementary material for: Stealth lipid polymer hybrid nanoparticles loaded with rutin for effective brain delivery – comparative study with the gold standard (Tween 80): optimization, characterization and biodistribution
Source: Drug Deliv. 2017 Dec 1;24(1):1874–90. doi: 10.1080/10717544.2017.1410263 (PMC8241138; doi:10.1080/10717544.2017.1410263)
Supplement: IDRD_Ishak_et_al_Supplemental_Content.docx [file IDRD_A_1410263_SM8006.docx]

| Response | EE% | PS | PDI |
| --- | --- | --- | --- |
| Suggested Model* | 2-factor interaction (2FI) | 2-factor interaction (2FI) | linear |
| Model Equation** | EE = +50.44 +10.45 X_1_+13.05 X_2_ -3.58 X_3_ +6.46 X_1_X_2_ | (PS)-1.37= +5.72x10^-4^-1.64*10^-4^ X_1_+6.85*10^-5^ X_2_+5.50*10^-5^ X_3_ -1.25*10^-4^ X_1_X_2_ -7.28*10^-5^ X_1_X_3_+1.50*10^-5^ X_2_X_3_ | PDI = +0.27 +0.10 X_1_ |
| R^2^ | 0.9856 | 0.9933 | 0.9273 |
| Adjusted R^2^ | 0.9760 | 0.9888 | 0.9091 |
| Predicted R^2^ | 0.9544 | 0.9787 | 0.8707 |
| Adequate Precision | 27.750 | 42.195 | 14.307 |

Table 1S. Statistical analysis of the 2-level full factorial design.

* The suggested models for all responses are found significant at *p*<0.0001.

** The equations of all models are represented with significant coded terms only at *p*<0.05 (with omitting non-significant ones).

Table 2S. Analysis of variance (ANOVA) results

| Terms | Responses | | | | | |
| --- | --- | --- | --- | --- | --- | --- |
|  | EE(%) | | PS | | PDI | |
|  | F-value | *p*-value | F-value | *p*-value | F-value | *p*-value |
| X_1_ | 200.80* | <0.0001 | 642.03* | <0.0001 | 150.62* | <0.0001 |
| X_2_ | 312.96* | <0.0001 | 111.57* | <0.0001 | 0.41^NS^ | 0.5340 |
| X_3_ | 23.51* | 0.0009 | 71.87* | <0.0001 | 1.94^NS^ | 0.1887 |
| X_1_X_2_ | 76.81* | <0.0001 | 370.65* | <0.0001 | --- | --- |
| X_1_X_3_ | 0.58^NS^ | 0.4671 | 126.07* | <0.0001 | --- | --- |
| X_2_X_3_ | 0.082^NS^ | 0.7810 | 5.37^**^ | 0.0457 | --- | --- |
| Lack of fit | 3.16^NS^ | 0.1133 | 0.53^NS^ | 0.4856 | 2.09^NS^ | 0.1742 |

X_1_: PLGA Amount (mg); X_2_: W_lecithin_/W_PLGA_ ratio; X_3_: Tween 80 concentration (%w/v).

*Significant at 1% probability (*p*<0.01), ** Significant at 5% probability (*p*<0.05), ^NS^ non-significant

Table 3S. QTPP for optimizing the CPP of LPH NPs.

| Response | Target | Reason |
| --- | --- | --- |
| RU EE | maximize | To warrant high drug loading into NPs and hence a sustained effect |
| PS | ≤ 250 nm | Smaller PS for long-circulating NPs not being detected by the reticuloendothelial system |
| PDI | ≤ 0.3 | Homogenous PS distribution |

Table 4S. Predicted and actual data of CQA of the optimized LPH NPs formulation

|  | RU EE (%) ±SD | PS (nm) ±SD | PDI ±SD |
| --- | --- | --- | --- |
| Predicted CQA | 69.13 | 241.89 | 0.3 |
| Actual CQA | 64.32 ±1.11 | 272.50 ±3.39 | 0.272 ±0.029 |
| Prediction error (%) | 7.48 | 11.23 | 10.29 |


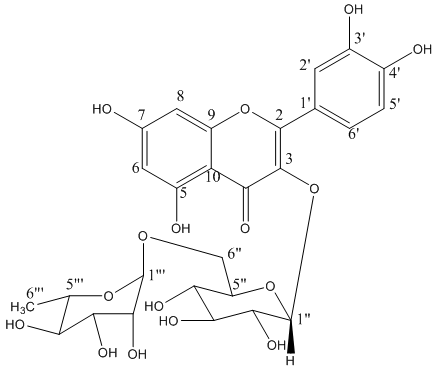


**Fig. 1S.** Chemical structure of quercetin-3-*O*-rutinoside (RU)

| 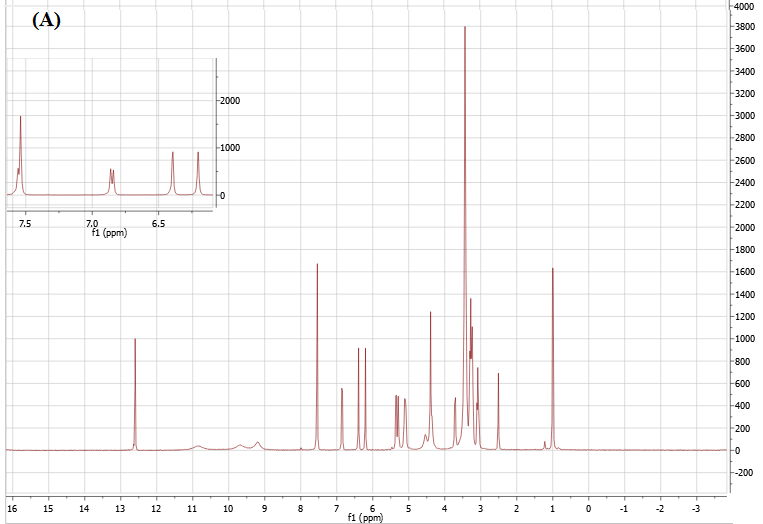 |
| --- |
| 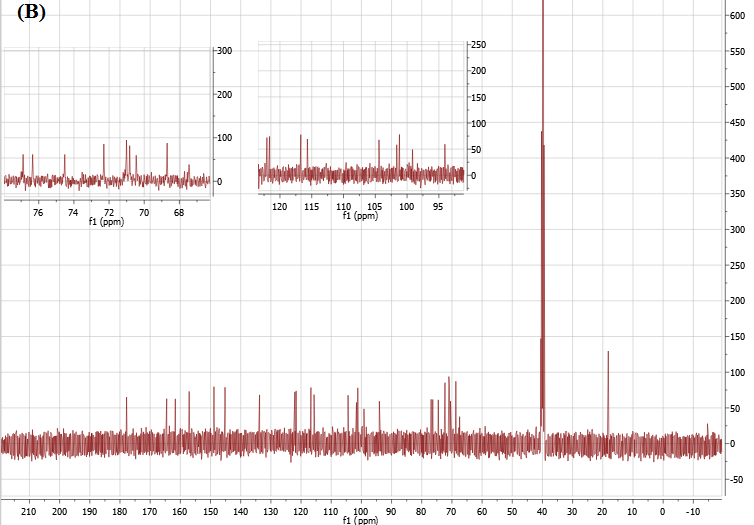 |

**Fig. 2S.** ^1^H-NMR (A) and ^13^C-NMR (B) of quercetin-3-rutinoside (RU)

**
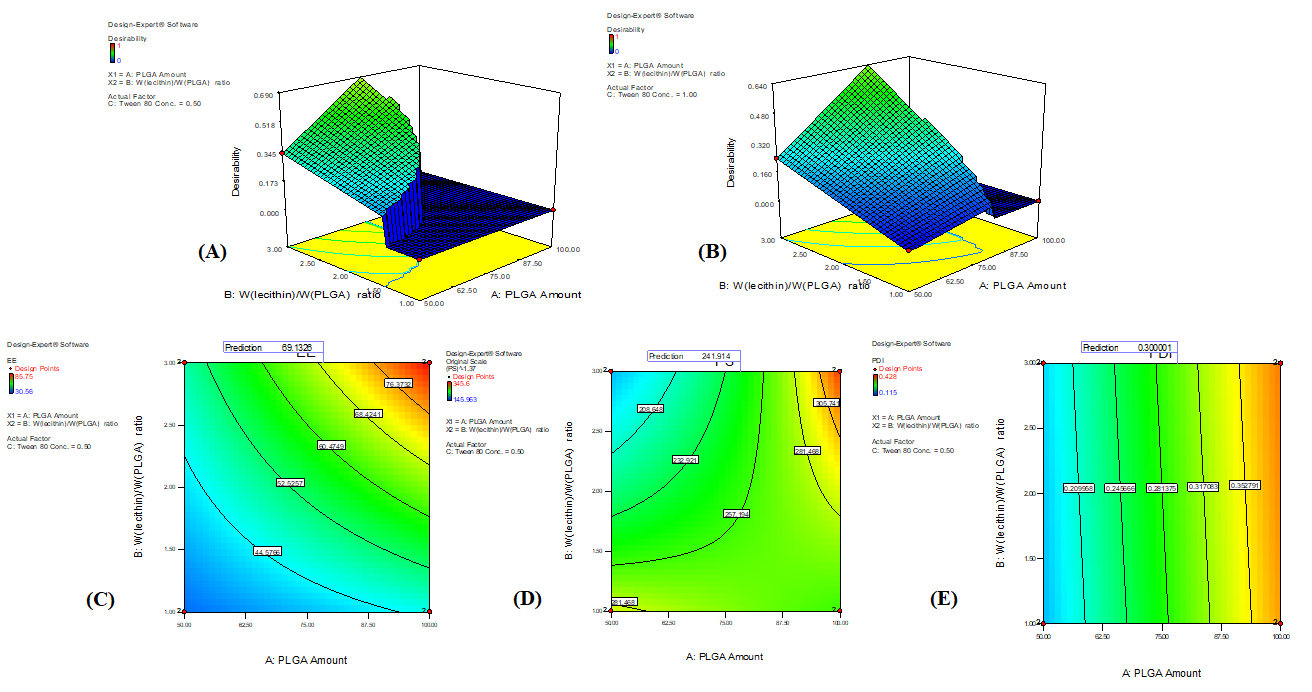
**

**Fig. 3S.** 3D-surface plots **(A, B)** illustrating the effect of PLGA amount (X_1_) and W_lecithin_/W_PLGA_ ratio (X_2_) on the desirability function (D) at 0.5 and 1% Tween 80 concentration (X_3_), respectively. Contour plots **(C-E)** showing the predicted values of EE, PS and PDI, respectively, at different X_1_ and X_2_ levels maintained at 0.5% Tween 80 concentration.
